# Dynamic changes of podocytes caused by fibroblast growth factor 2 in culture

**DOI:** 10.1007/s00441-021-03511-x

**Published:** 2021-07-26

**Authors:** Eishin Yaoita, Masaaki Nameta, Yutaka Yoshida, Hidehiko Fujinaka

**Affiliations:** 1 Department of Structural Pathology, Kidney Research Center, Niigata University Graduate School of Medical and Dental Sciences, Niigata, Japan; 2 Cooperative Laboratory of Electron Microscopy, Niigata University, Niigata, Japan; 3 Department of Clinical Research, Niigata National Hospital, Kashiwazaki, Japan

**Keywords:** FGF2, Podocyte, Culture, Motility, Slit diaphragm

## Abstract

Fibroblast growth factor 2 (FGF2) augments podocyte injury, which induces glomerulosclerosis, although the mechanisms remain obscure. In this study, we investigated the effects of FGF2 on cultured podocytes with interdigitating cell processes in rats. After 48 h incubation with FGF2 dynamic changes in the shape of primary processes and cell bodies of podocytes resulted in the loss of interdigitation, which was clearly shown by time-lapse photography. FGF2 reduced the gene expressions of constituents of the slit diaphragm, inflections of intercellular junctions positive for nephrin, and the width of the intercellular space. Immunostaining for the proliferation marker Ki-67 was rarely seen and weakly stained in the control without FGF2, whereas intensely stained cells were frequently found in the presence of FGF2. Binucleation and cell division were also observed, although no significant increase in cell number was shown. An in vitro scratch assay revealed that FGF2 enhanced migration of podocytes. These findings show that FGF2 makes podocytes to transition from the quiescent state into the cell cycle and change their morphology due to enhanced motility, and that the culture system in this study is useful for analyzing the pathological changes of podocytes in vivo.

**Supplementary Information:**

The online version contains supplementary material available at 10.1007/s00441-021-03511-x.

## Introduction

Visceral glomerular epithelial cells in the kidney, which are referred to as podocytes, reside in a quiescent state and reveal no evidence of proliferation (Pabst and Sterzel 1983). Podocytes interdigitate with each other in an elaborate morphology of cell bodies, primary processes, ridge-like prominences, and foot processes (Burghardt et al. 2015; Ichimura et al. 2015). Additionally, they possess a unique intercellular junction called the slit diaphragm, which plays a crucial role in the glomerular filtration barrier. Clinical and experimental studies have provided evidence that podocyte injury leads to focal and segmental glomerulosclerosis (FSGS) and eventually, glomerular tuft destruction, which are common histological findings in the progression of chronic renal diseases (Pavenstädt et al. 2003).

Daily injections of fibroblast growth factor 2 (FGF2) into rats for several weeks induce remarkable morphological changes that indicated mitosis and injury of podocytes (Floege et al. 1995; Kriz et al. 1995; Mazué et al. 1993). These changes are followed by the development of widespread FSGS (Kriz et al. 1995). Furthermore, it was suggested in a study of passive Heyman nephritis with FGF2 administration that FGF2 may enhance podocyte damage (Floege et al. 1995). Podocyte lesions are assumed to be caused by the direct effects of FGF2 and adaptation to the environment, such as glomerular hypertrophy and hyperfiltration. FGF2 has pleiotropic roles in several cell types and tissues; it is an angiogenic and survival factor which is involved in cell proliferation, differentiation, and migration, as well as a variety of developmental processes (Moscatelli et al. 1986; Okada-Ban et al. 2000). The direct effects of FGF2 on podocytes are poorly understood. A previous study reported that FGF2 promoted cell proliferation of cultured glomerular epithelial cells (Takeuchi et al. 1992). However, the cells used in the study exhibited characteristics different from those of podocytes in vivo. First, cells cultured in the presence of fetal bovine serum (FBS) actively proliferated in the control without FGF2, whereas podocytes in vivo resided in a quiescent state. Second, they possessed a simple polygonal morphology with a cobblestone-like appearance, whereas the counterparts in vivo formed an elaborate morphology with interdigitation. Third, polygonal cells likely came from parietal epithelial cells of Bowman capsule but not from podocytes (Yaoita et al. 1991; Oyama et al. 2020). Thus, to elucidate the effects of FGF2 on podocytes, it is necessary to investigate cultured cells that exhibit phenotypes that are similar to those of podocytes in vivo.

Recently, we established culture conditions that restore podocytes to the specific morphology with interdigitating cell processes (Yaoita et al. 2018). The serum-free culture medium contained all-trans-retinoic acid (ATRA). Under this culture condition, cells rarely divided, although several cells were binucleate cells when they grew out from isolated glomeruli (Katsuya et al. 2006). In this study, the effects of FGF2 on podocytes were investigated to compare cultured cells that share features with podocytes in vivo, which demonstrated dynamic changes in podocyte morphology and gene expressions induced by FGF2.

## Materials and methods

### Animals

Male Sprague Dawley rats were purchased from Charles River Laboratories Japan, Inc. (Atsugi, Japan), and were 9 weeks old when used in the experiments.

Culture for podocytes (Online Resource 1).

Rat glomeruli were isolated and cultured to obtain outgrowths of podocytes following a previously described protocol with minor modifications (Yaoita et al. 2018; Oyama et al. 2020). Kidney cortices perfused with iron powder were digested with collagenase and passed through a 100-μm cell strainer. Glomeruli containing magnetic particles were collected using a magnet and were cultured. After 3 days of culturing, cellular outgrowths from glomeruli were detached using a non-enzymatic cell dissociation solution. The cell suspension was filtered through a 40-μm cell strainer to remove remaining glomerular core. Then, the cells were cultured in the same medium on glass slides printed with a highly water-repellent mark with wells 5 mm in diameter (TF1205M, Matsunami Glass Ind., Ltd., Osaka, Japan). The wells were previously coated with laminin-521 by adding 15 μl of laminin-521 (20 μg/ml) to each well, then incubating at 4 °C overnight. Cells were cultured at a density of 1.4 × 10^4^ cells per well. After 6 h, most of the cells had attached to the glass slides, and the culture medium was changed to D-MEM/F-12 containing 0.5% Insulin-Transferrin-Selenium-A supplement (ITS), 0.5% FBS, 0.2 mg/ml dextran sulfate (DS), 0.2 μM ATRA, and antibiotic solution. After 24 h, the medium was replaced with D-MEM/F-12 containing 0.5% ITS, 0.2 μM ATRA, and antibiotic solution. After 6 days of culturing, FGF2 (recombinant human Fibroblast Growth Factor 2, Oriental Yeast Co. Ltd., Tokyo, Japan) was added to the culture media in concentrations ranging from 5 to 100 ng/ml. After culturing for another 2 days, cells were processed for morphological or immunohistochemical analysis or RNA extraction.

### Quantitative RT-PCR analysis

RNA extraction, cDNA synthesis, and real-time polymerase chain reaction (RT-PCR) were performed as previously described (Yaoita et al. 2014; Oyama et al. 2020). To measure the amount of specific mRNA in each sample, a standard curve was generated for each run using serial dilutions of cDNA from isolated glomeruli. The gene expression was normalized to levels of *Gapdh* mRNA. To quantify mRNA, each gene evaluated by RT-PCR was expressed relative to that in the isolated glomeruli, which was assigned a value of 1.0. Table 1 shows the sequences of the primers.

**Table 1:** Primers sets for PCR analysis

	Accession number	Primers (forward, reverse)
*Gapdh*	NM_017008	taaagggcatcctgggctacact, ttactccttggaggccatgtagg
*Nphs1* (nephrin)	NM_022628	tggttcgtcttgtcgtccga, ctggatgttggtgtggtcag
*Kirrel1* (Neph1)	NM_207606	caaagtcgggagcaccaat, ttcccaacccagacacaagtg
*Nphs2* (podocin)	NM_130828	agcagtctagctcatgtgtcca, gcagccgtacatccttaatttc

### Confocal immunofluorescence microscopy and electron microscopy

Indirect immunofluorescence microscopy and transmission or scanning electron microscopy were performed as previously described (Yaoita et al. 1991, 2001). The following antibodies used for immunofluorescence microscopy included mouse monoclonal anti-vimentin antibody (clone V9; Sigma, Saint Louis, MO, USA), mouse monoclonal anti-desmin antibody (clone D33, DakoCytomation Denmark A/S, Glostrup, Denmark), rabbit monoclonal anti-vimentin antibody (ab 92,547, Abcam plc, Cambridge, UK), rabbit monoclonal anti-Ki67 antibody (ab16667, Abcam plc), guinea pig polyclonal anti-nephrin antibody (Progen Biotechnik GmbH, Heidelberg, Germany), and rabbit polyclonal anti-podocin antibody (Immuno-Biological Laboratories Co., Ltd., Gunma, Japan). Normal rabbit serum and mouse monoclonal anti-keyhole limpet hemocyanin antibodies (clone: #11,711, R&D Systems, Inc., Minneapolis, MN, USA) were used as negative controls.

The quantification of the nephrin-positive area was performed according to the protocol described by Mizukami et al. with minor modifications (Mizukami et al. 2016). Briefly, immunofluorescence microscopic images were transferred into Photoshop software (v 22.3.1, Adobe Systems Inc, San Jose, CA, USA). After removing background fluorescence using “Image Adjustment,” the nephrin-positive lines were highlighted with “fuzziness” at 100 and the pixels that composed linear immunofluorescence were counted using the pixel measurement function of the Photoshop software (Online Resource 2). The area of nephrin-positive lines was expressed as the ratio of the pixel number of the nephrin-positive lines to the pixel number of the total image.

Using sections double-labeled with anti-vimentin antibody and 4′,6-diamidino-2-phenylindole (DAPI), the cells were counted and distinguished as single-nucleated or multinucleated cells. The base area per cell was measured by dividing the area of one image by the number of cells occupying it.

### In vitro scratch assay

The cells on the glass slide were scratched using yellow tips on the seventh day after the subculture and then were cultured in the presence of FGF2 for another 24 h. The width of cell-free space (m) and the width between the boundaries on both sides of the scratch between shape-changed cells and unchanged cells (n) were measured under a phase-contrast microscope. The migration distance was expressed as (*n* − *m*)/2.

### Statistical analysis

The results were expressed as mean ± standard deviation. Statistical analyses were performed using GraphPad Prism software (version 9) and consisted of 1-way analysis of variance followed by Dunnet’s multiple comparison test when comparing > 2 experimental groups or Mann–Whitney *U*-test when comparing 2 experimental groups. *P* < 0.05 were considered statistically significant.

## Results

### Phase-contrast microscopic observation

**Fig. 1:**
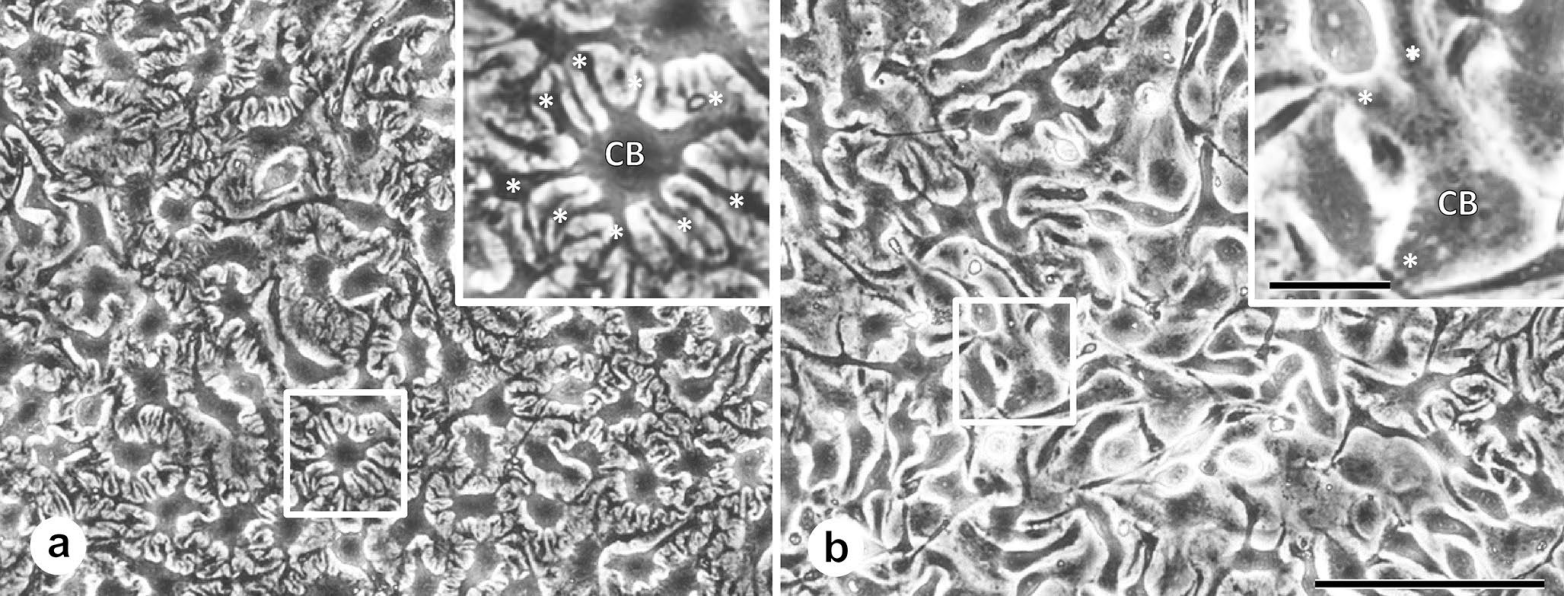
Phase-contrast microscopic images of cultured podocytes after 48 h incubation without FGF2 (**a**) and with 20 ng/ml FGF2 (**b**). Primary processes (asterisks in inset) extended from the cell body (CB in inset), branched and interdigitated with each other (**a**), whereas most branching cell processes were lost, and the number of primary processes decreased in the presence of FGF2 (**b**). Scale bar = 100 μm in **b**, 20 μm in insert in **b**

Six days after subculture, podocytes projected several primary branching processes and interdigitated among one other like their counterparts in vivo (Online Resource 3). Morphologies did not change on day 8 (Fig. 1a). Time-lapse photography revealed slight movements inside the bodies of each cell and repeating the formations and disappearance of branches at the peripheries of primary processes from day 6 to day 8 (Online Resource 4). Incubation with FGF2 during the 2-day period caused morphological changes of the primary processes and cell bodies. In 10 ng/ml of FGF2, primary processes branched less (Online Resource 5). The higher concentration of FGF2 (20 ng/ml) caused noticeable changes (Fig. 1b). Many cells lost primary process branching, and the number of primary processes decreased significantly. The number of primary processes extending directly from the cell body was 6.88 ± 1.83 in the controls and 3.09 ± 1.24 in the 20 ng/ml FGF2-incubated groups (*P* < 0.01, *n* = 6). Time-lapse photography showed these dynamic changes clearly (Online Resource 6).

### Gene expressions of constituents of the slit diaphragm

**Fig. 2:**
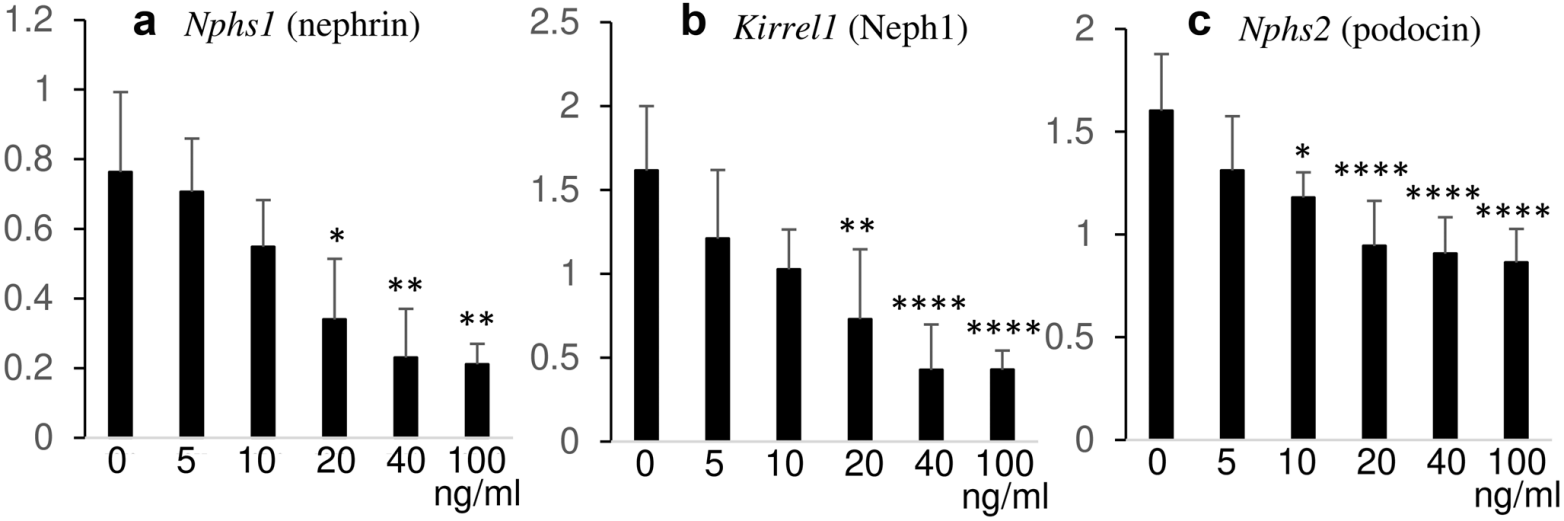
Gene expressions of constituents of the slit diaphragm: *Nphs1* (nephrin) (**a**), *Kirrel1* (Neph1) (**b**), and *Nphs2* (podocin) (**c**). Values represent mean ± s.d. of six independent experiments. ∗ *P* < 0.05, ∗  ∗ *P* < 0.01, and ∗  ∗  ∗  ∗ *P* < 0.0001 versus respective control without FGF2 and derived from 1-way analysis of variance with Dunnet’s multiple comparisons test

Because morphological alterations of podocytes are usually accompanied by the decrease or disappearance of the slit diaphragm, gene expression levels related to the slit diaphragm were examined via RT-PCR (Fig. 2). Primary constituents of the slit diaphragm showed significantly decreased *Nphs1* (nephrin), *Nphs2* (podocin), and *Kirrel1* (Neph1) expressions in a dose-dependent manner of FGF2.

### Immunofluorescence microscopic and transmission electron microscopic observations of the slit diaphragm

**Fig. 3:**
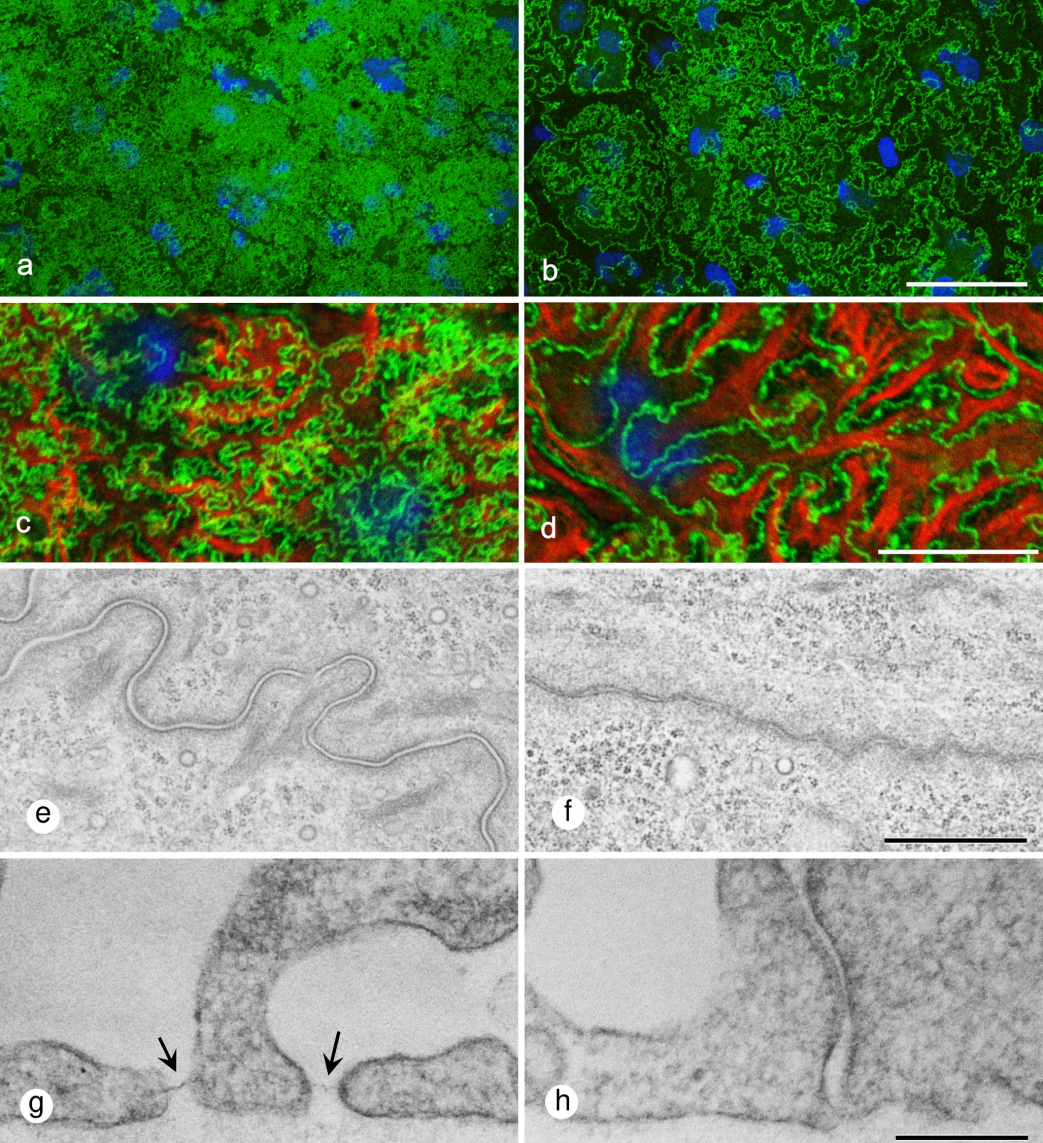
Immunofluorescence microscopic (**a**–**d**) and transmission electron microscopic images (**e**–**h**) showing the slit diaphragm after 48 h incubation without FGF2 (**a**, **c**, **e**, **g**) and with 40 ng/ml FGF2 (**b**, **d**, **f**, **h**). Immunofluorescence for nephrin (green) formed extremely fine curves (**a**). The degree of inflections was much less in podocytes incubated with 40 ng/ml FGF2 than those of the control without FGF2 (**b**). Nuclei are colored blue with DAPI. In the control, the immunofluorescence for nephrin (green) often overlapped with vimentin-positive primary processes (red) (**c**). After 48 h incubation with FGF2, nephrin was confined between primary processes (**d**). Electron microscopy of the cross sections parallel to the glass slide revealed that the intercellular junctions including the slit diaphragm in the control exhibited smooth curves (**e**). The intercellular space at the slit junctions was relatively uniform and approximately 30–40 nm wide (**e**), which was narrow and not uniform in podocytes incubated with FGF2 (**f**). Cross sections perpendicular to the glass slide also showed a 40-nm-wide slit diaphragm (arrows) in the control (**g**) and narrower intercellular space in FGF2-treated podocyte (**h**). Scale bar = 50 μm in **a** and **b**, 20 μm in **c** and **d**, 1 μm in **e** and **f**, and 200 nm in **g** and **h**

The distribution of nephrin and podocin, which are the main constituents of the slit diaphragm, was compared with and without FGF2. In the control without FGF2, immunostaining for nephrin formed extremely fine curves (Fig. 3a). The curve inflections were so fine that some parts of staining looked like a solid color. Incubation with FGF2 reduced the inflections of nephrin-positive lines (Fig. 3b). The area of nephrin-positive lines was significantly smaller in the FGF2-incubated groups than the controls (0.623 ± 0.029 for controls vs. 0.239 ± 0.057 for 40 ng/ml FGF2 groups; *P* < 0.01, *n* = 6). Double immunofluorescence microscopy for nephrin and vimentin revealed that the nephrin-positive curve lines were present both between and under the vimentin-positive primary processes in the controls (Fig. 3c), while the lines were confined only between primary processes after 48 h incubation with FGF2 (Fig. 3d). Podocin staining showed the same result as that of nephrin (data not shown). Electron microscopy showed smooth curves of intercellular junctions, which included the slit diaphragm, with a relatively constant width of the intercellular space in the control (Fig. 3e, g). The intercellular space was narrow and non-uniform in the presence of FGF2 (Fig. 3f, h).

### Immunofluorescence microscopic observation for the entry into cell cycle

**Fig. 4:**
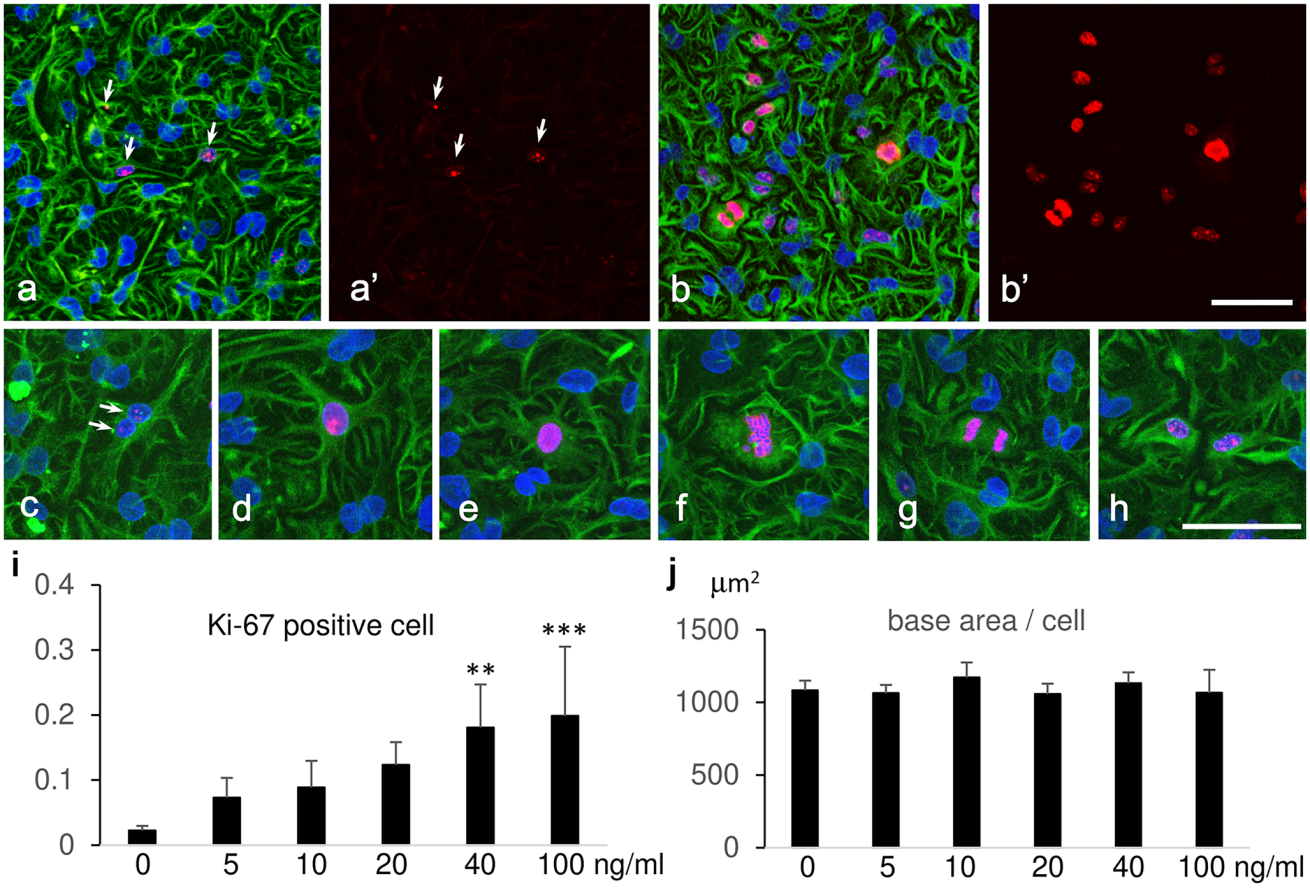
Double-labeled immunofluorescence microscopic images showing Ki-67 and vimentin after 48 h incubation without FGF2 (**a**, **a′**, **c**) and with 40 ng/ml FGF2 (**b**, **b′**, **d**–**h**). Ki-67 (red) was localized in nuclei while vimentin (green) was localized in cell bodies and cell processes. Nuclei are colored blue with DAPI. Vimentin staining revealed the culture at confluence. Ki-67 staining looked like small dots in the control without FGF2 (arrows in **a**, **a′**, **c**). FGF2 increased the staining intensity and the number of Ki-67-positive cells (**b**, **b′**); **c**–**h** show Ki-67-positive cells at high magnification; **f**, **g**, and h show cells from metaphase to telophase. Scale bar = 100 μm in **b′** and 50 μm in **h**; **i** indicates the ratio of the number of Ki-67-positive cells to the total number of cells; **j** indicates the base area per cell. The X-axis shows the FGF2 concentration. Values represent mean ± s.d. of five independent experiments. ∗  ∗ *P* < 0.01, and ∗  ∗  ∗ *P* < 0.001 versus respective control without FGF2 and derived from 1-way analysis of variance with Dunnet’s multiple comparisons test

**Fig. 5:**
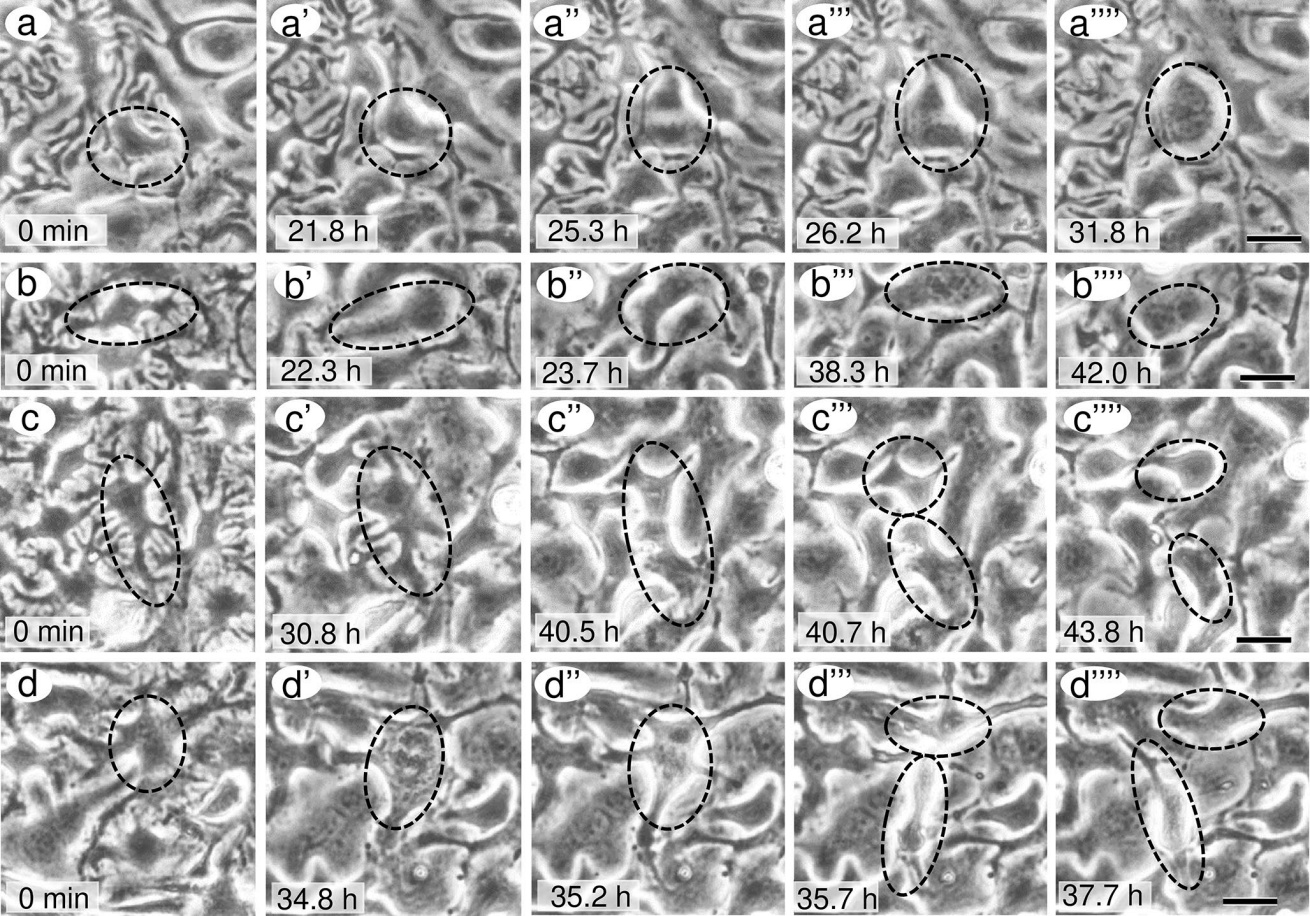
Phase-contrast microscopic images of cultured podocytes during 48 h incubation with 40 ng/ml FGF2. Binucleation (**a**–**a″″**, **b**–**b″″**) and cell division (**c**–**c″″**, **d**–**d″″**) were observed. Dotted circles follow the changes in the same cell over time. The numbers indicate the elapsed time after the addition of FGF2. Scale bar = 20 μm

Mitotic figures in podocytes were found in FGF2-treated rats, so Ki-67 staining was examined by immunofluorescence microscopy. Ki-67 is a proliferation marker, as it is present during all active phases of the cell cycle but is absent in quiescent cells (Bruno and Darzynkiewicz 1992; Scholzen and Gerdes 2000). Ki-67-positive cells were rare in the control without FGF2. Their staining was punctate and weak (Fig. 4a, a′, c). After the incubation of FGF2, the staining intensity increased conspicuously (Fig. 4b, b′, d–h). Some cells were clearly in metaphase or anaphase (Fig. 4f–h). The positive cells increased in number in a dose dependent manner of FGF2 (Fig. 4i). Despite the increased number of Ki-67 positive cells, the base area per cell did not show a significant change (Fig. 4j), which indicated that there was no significant increase in cell number. The proportion of single-nucleated cells tended to decrease as the FGF2 concentration increased, although it was not significant (control 35.8% ± 4.1%, 20 ng/ml FGF 31.3% ± 4.0%, 40 ng/ml FGF 29.5% ± 2.8%, 100 ng/ml 26.5% ± 5.1%). Time-lapse photographic images were examined in detail to see if cells that entered into mitosis underwent cell division besides binucleation (Fig. 5a, b). Consequently, some of the cells underwent cell division (Fig. 5c, d). Because the number of cells observed by time-lapse photography was too small, the proportion of cells that underwent cell division could not be determined. During and after mitosis, the cells changed their shape to lose branches of primary processes and move considerably.

### In vitro scratch assay for cell migration

**Fig. 6:**
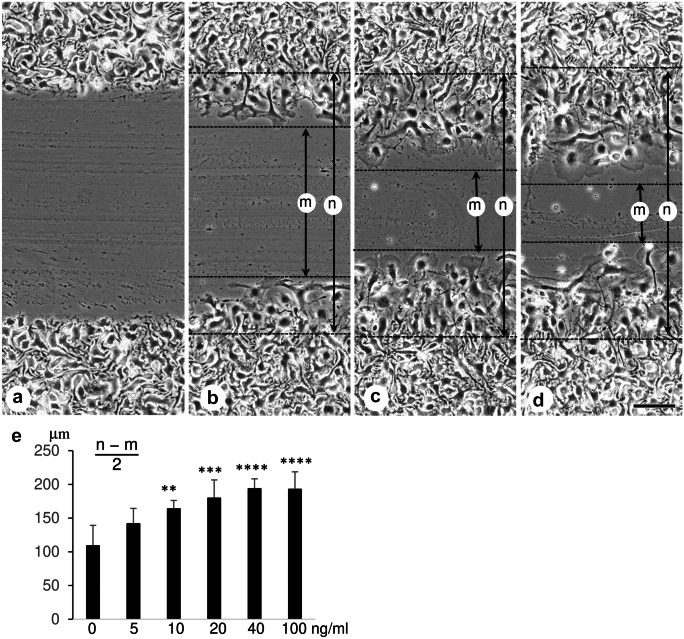
Phase-contrast microscopic images immediately after scratching (**a**) and images of cultured podocytes 24 h after scratching incubating without FGF2 (**b**) and with 10 ng/ml (**c**) and 40 ng/ml (**d**) FGF2. Scale bar = 100 μm; (**e**) indicates the migration distance of podocytes 24 h after scratching; *x*-axis shows the FGF2 concentration; *m* is the width of cell-free space; *n* is the width of the boundaries on both sides between shape-changed cells and unchanged cells. Values represent mean ± s.d. of six independent experiments. ∗  ∗ *P* < 0.01, ∗  ∗  ∗ *P* < 0.001, and ∗  ∗  ∗  ∗ *P* < 0.0001 versus respective control without FGF2 and derived from 1-way analysis of variance with Dunnet’s multiple comparisons test

The effect of FGF2 on migration was investigated, as phase-contrast microscopic observations showed substantial changes and movements. The migration distance was measured 24 h after the scratch assay. Time-lapse photography revealed that three to five layers of cells adjacent to the scratch were involved in migration in 24 h (Online Resource 7). FGF2 significantly increased the migration distance (Fig. 6).

## Discussion

In the present study, the effects of FGF2 on podocytes were investigated in the culture. Consequently, it was found that FGF2 caused significant changes in podocyte morphologies and gene expressions in a dose-dependent manner. The remarkable findings are time-lapse microscopic observations of interdigitating podocytes and reproduction of some of pathological changes in vivo by culture.

In the control without FGF2, slight but active movements were observed at the peripheries of the primary processes and inside the cell bodies. In the presence of FGF2, podocytes actively changed primary processes and cell bodies. Time-lapse photography demonstrated these dynamic changes clearly. Additionally, in vitro scratch assays showed that FGF2 increased podocyte cell migration. These findings indicate that FGF2 enhances podocyte motility.

FGF2 caused decreases in gene expressions of constituents of the slit diaphragm, the disappearance of branching primary processes, and the narrowing of the intercellular space. All these changes are shared with podocytes in vivo in proteinuric states (Ichimura et al. 2019; Inokuchi et al. 1996; Pavenstädt et al. 2003). It is proper to assume that the simplification of curves positive for nephrin in immunofluorescence microscopy corresponded to the effacement of foot processes. Thus, cultured podocytes in this study reproduced pathological changes in vivo. It is interesting to elucidate how enhanced motility is associated with these pathological changes.

Immunofluorescence microscopy for Ki-67, which is a marker for cell proliferation, showed that FGF2 increased the number of Ki67-positive cells in a dose-dependent manner. Ki-67 is absent in quiescent cells (Bruno and Darzynkiewicz 1992; Scholzen and Gerdes 2000). Only 2% of cultured cells exhibited weak and punctate immunofluorescence for Ki-67 in the control, which indicated that most of the cells were in the quiescent state. FGF2 significantly increased Ki-67 staining intensity and positive cells. The findings demonstrated that FGF2 caused quiescent podocytes enter the cell cycle.

The incubation with FGF2 did not change the base area per cell. Interestingly, its area is comparable with the glomerular surface area covered by one podocyte of the human and rat (Hishiki et al. 1999, 2001; Pagtalunan et al. 1997). Because the cultures were always confluent, the stable base area meant that the number of cells did not increase significantly. As approximately 36% of cells had a single nucleus in the control, more than half of the cells were binucleated or multinucleated. FGF2 tended to decrease the proportion of single-nucleated cells, which is consistent with the fact that the number of cells did not increase. These findings indicate that podocytes are prone to binucleation rather than cell division during culturing, as well as in vivo, although time-lapse photography revealed cell divisions occurring in very few cells. In the cases of binucleation and cell division, cells changed their shape actively and move considerably during and after mitosis. Entry into the cell cycle may have caused enhanced mobility, but further experiments are necessary to elucidate this. Moreover, the proportion of cells with changing morphologies appeared to be higher than that of Ki-67 positive cells.

Rats treated with FGF2 exhibit FSGS. Podocytes in the rats exhibit several changes that were not observed in this study. The changes include hypertrophy, cell body attenuation, pseudocyst formation, detachment from the glomerular basement membrane, and enhanced desmin staining (Kriz et al. 1995). Apparent hypertrophy was observed in the migration after scratch, although significant hypertrophy was not recognized in the incubation with FGF2, because the base area per cell did not significantly change, and the height of the cells could not be measured. Neither cell body attenuation nor pseudocyst formation was found via electron microscopy. There was no cell-free space that was indicated by detachment. The incubation with FGF2 did not cause significantly increased expressions of desmin in gene or in immunostaining (data not shown). The difference in the culture versus in vivo is the absence of the glomerular filtration pressure, which produces glomerular filtration and an expansile force of the basement membrane (Kriz et al. 1995, 2015). Therefore, the above changes in vivo are likely the result of adapting to a changing environment in vivo, such as glomerular hypertrophy or hyperfiltration. Thus, the culture system utilized in this study will elucidate the direct effect of causative substances distinguished from podocyte responses to the environment in vivo.

## Supplementary Information

Below is the link to the electronic supplementary material.

Supplementary file1 Online Resource 1.pdf Experimental protocol. ITS, Insulin-Transferrin-Selenium-A Supplement; FBS, fetal bovine serum; DS, dextran sulfate; ATRA, all-trans-retinoic acid. (PDF 35 KB)

Supplementary file2 Online Resource 2.pdf Image analysis example of quantifying the nephrin-positive area using Photoshop. (PDF 1255 KB)

Supplementary file3 Online Resource 3.pdf Scanning electron microscopic images of podocytes in culture (a, c) and in vivo (b,d) and phase contrast microscopic images of podocytes in culture (e, f). Cell bodies (CB) and primary processes (asterisks) were identified in cultured podocytes by corresponding with images of podocytes in vivo. The primary processes interdigitated with those of adjacent cells (c, f). Foot processes were hidden under the primary processes (c) or were too small to recognize under the phase contrast microscope (f). (PDF 439 KB)

Supplementary file4 Online Resource 4.mp4 Time-lapse photography of podocytes for 48 h from day 6 to day 8 in subculture. The primary process repeats the formation and disappearance of branches in the periphery (red circle). Images were captured every 10 min and the movie is viewed at 15 frames/s. Scale bar = 50 μm. (MP4 69273 KB)

Supplementary file5 Online Resource 5.mp4 Time-lapse photography of podocytes in the presence of 10 ng/ml FGF2 for 48 h from day 6 to day 8 in subculture. Images were captured every 10 min and the movie is viewed at 15 frames/s. Scale bar = 50 μm. (MP4 64466 KB)

Supplementary file6 Online Resource 6.mp4 Time-lapse photography of podocytes in the presence of 40 ng/ml FGF2 for 48 h from day 6 to day 8 in subculture. Images were captured every 10 min and the movie is viewed at 15 frames/s. Scale bar = 50 μm. (MP4 72904 KB)

Supplementary file7 Online Resource 7.mp4 Time-lapse photography of podocyte migration after scratch in the presence of 20 ng/ml FGF2 for 24 h from day 7 to day 8 in subculture. Images were captured every 10 min and the movie is viewed at 15 frames/s. Scale bar = 50 μm. (MP4 32603 KB)
