# Supplementary material for: Dynamic changes of podocytes caused by fibroblast growth factor 2 in culture
Source: Cell Tissue Res. 2021 Jul 26;386(1):117–26. doi: 10.1007/s00441-021-03511-x (PMC8526483; doi:10.1007/s00441-021-03511-x)
Supplement: Supplementary file 1 — Supplementary file1 Online Resource 1.pdf Experimental protocol. ITS, Insulin-Transferrin-Selenium-A Supplement; FBS, fetal bovine serum; DS, dextran sulfate; ATRA, all-trans-retinoic acid. (PDF 35 KB) [file 441_2021_3511_MOESM1_ESM.pdf]

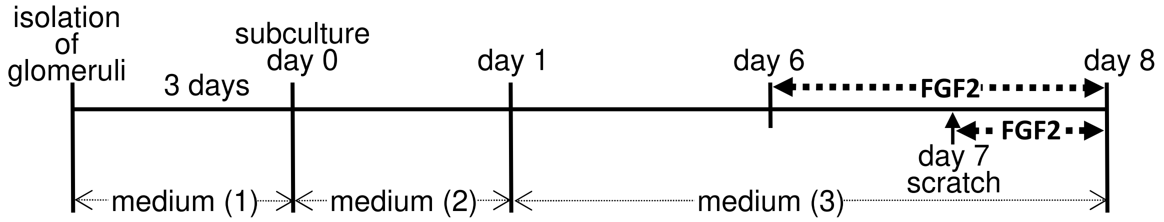

Medium (1); DMEM/F-12 supplemented with 0.5% ITS, 5% FBS
Medium (2); DMEM/F-12 supplemented with 0.5% ITS, 0.5% FBS, 0.2 mg/ml DS, 0.2 μM ATRA
Medium (3); DMEM/F-12 supplemented with 0.5% ITS, 0.2 μM ATRA

Online Resource Online 1 Experimental protocol. ITS, Insulin-Transferrin-Selenium-A Supplement; FBS, fetal bovine serum; DS, dextran sulfate; ATRA, all-trans-retinoic acid.

Article title: Dynamic changes of podocytes caused by fibroblast growth factor 2 in culture
Journal name: Cell and Tissue Research
Author names: Eishin Yaoita, Masaaki Nameta, Yutaka Yoshida, Hidehiko Fujinaka
Affiliation and e-mail address of the corresponding author : Department of Structural Pathology, Kidney Research Center, Niigata University Graduate School of Medical and Dental Sciences, Niigata, Japan, eyaoita@med.niigata-u.ac.jp
