# Supplementary material for: Dynamic changes of podocytes caused by fibroblast growth factor 2 in culture
Source: Cell Tissue Res. 2021 Jul 26;386(1):117–26. doi: 10.1007/s00441-021-03511-x (PMC8526483; doi:10.1007/s00441-021-03511-x)
Supplement: Supplementary file 2 — Supplementary file2 Online Resource 2.pdf Image analysis example of quantifying the nephrin-positive area using Photoshop. (PDF 1255 KB) [file 441_2021_3511_MOESM2_ESM.pdf]

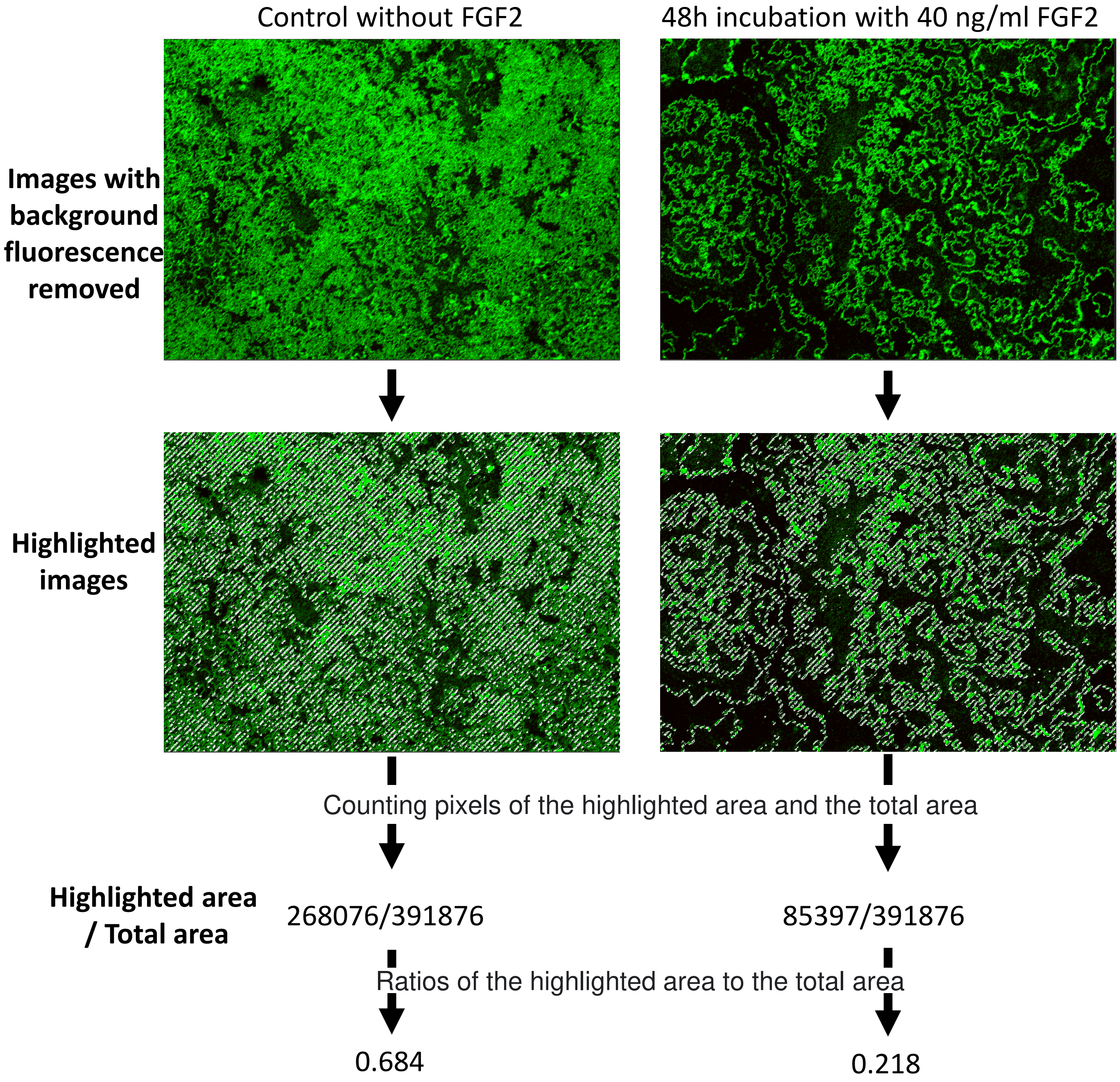

Online Resource 2 Image analysis example of quantifying the nephrin-positive area using Photoshop.

Article title: Dynamic changes of podocytes caused by fibroblast growth factor 2 in culture
Journal name: Cell and Tissue Research
Author names: Eishin Yaoita, Masaaki Nameta, Yutaka Yoshida, Hidehiko Fujinaka
Affiliation and e-mail address of the corresponding author : Department of Structural Pathology, Kidney Research Center, Niigata University Graduate School of Medical and Dental Sciences, Niigata, Japan, eyaoita@med.niigata-u.ac.jp
