# Supplementary material for: Dynamic changes of podocytes caused by fibroblast growth factor 2 in culture
Source: Cell Tissue Res. 2021 Jul 26;386(1):117–26. doi: 10.1007/s00441-021-03511-x (PMC8526483; doi:10.1007/s00441-021-03511-x)
Supplement: Supplementary file 3 — Supplementary file3 Online Resource 3.pdf Scanning electron microscopic images of podocytes in culture (a, c) and in vivo (b,d) and phase contrast microscopic images of podocytes in culture (e, f). Cell bodies (CB) and primary processes (asterisks) were identified in cultured podocytes by corresponding with images of podocytes in vivo. The primary processes interdigitated with those of adjacent cells (c, f). Foot processes were hidden under the primary processes (c) or were too small to recognize under the phase contrast microscope (f). (PDF 439 KB) [file 441_2021_3511_MOESM3_ESM.pdf]

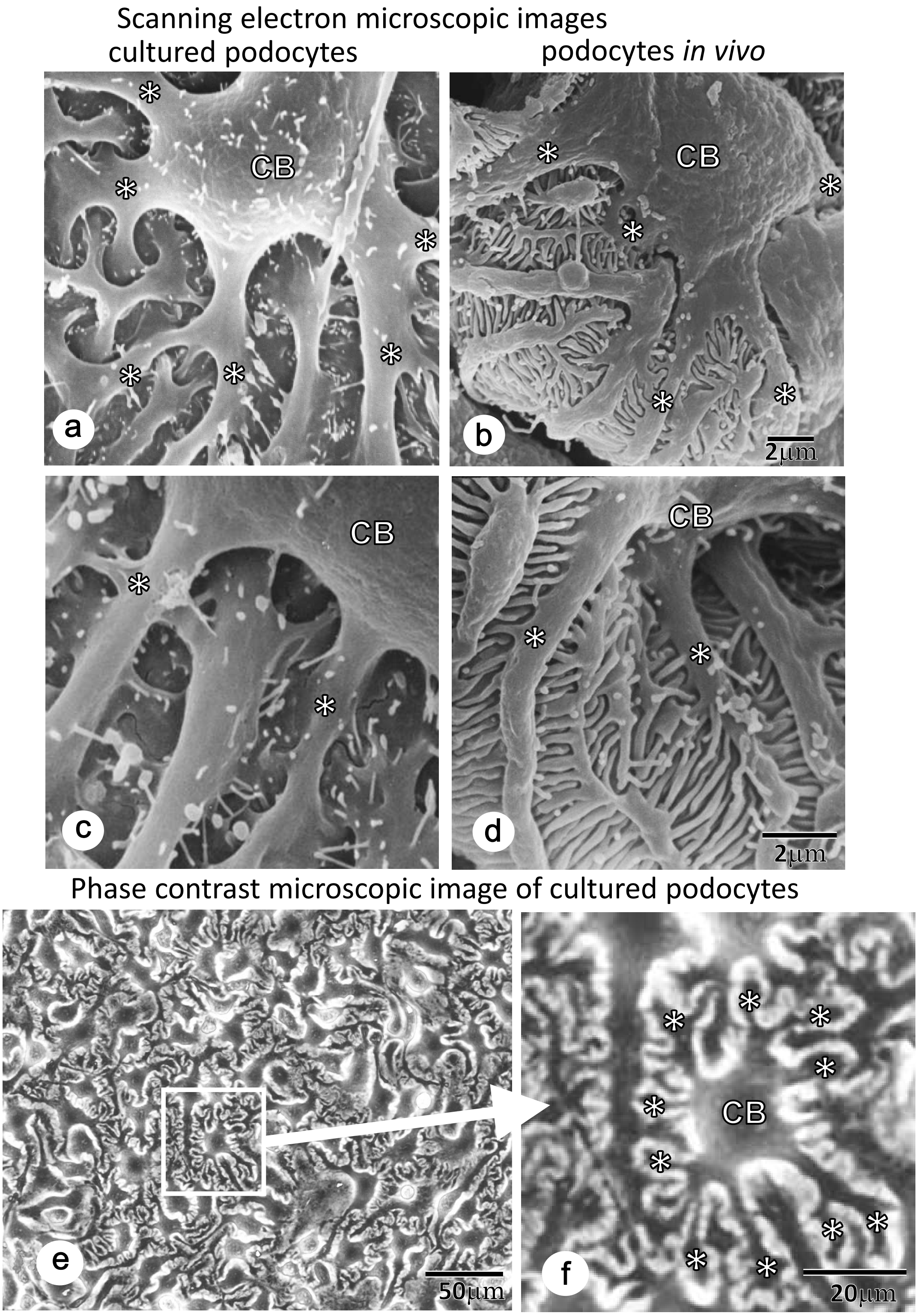

Online Resource 3 Scanning electron microscopic images of podocytes in culture (a, c) and *in vivo* (b,d) and phase contrast microscopic images of podocytes in culture (e, f). Cell bodies (CB) and primary processes (asterisks) were identified in cultured podocytes by corresponding with images of podocytes *in vivo*. The primary processes interdigitated with those of adjacent cells (c, f). Foot processes were hidden under the primary processes (c) or were too small to recognize (f).

Article title: Dynamic changes of podocytes caused by fibroblast growth factor 2 in culture
Journal name: Cell and Tissue Research
Author names: Eishin Yaoita, Masaaki Nameta, Yutaka Yoshida, Hidehiko Fujinaka
Affiliation and e-mail address of the corresponding author : Department of Structural Pathology, Kidney Research Center, Niigata University Graduate School of Medical and Dental Sciences, Niigata, Japan, eyaoita@med.niigata-u.ac.jp
